# Linear IgA Bullous Dermatosis

**Published:** 2013-07-02

**Authors:** Sean Chen, Peter Mattei, Max Fischer, Joshua D. Gay, Stephen M. Milner, Leigh Ann Price

**Affiliations:** ^a^Department of Dermatology and Dermatopathology; ^b^Department of Plastic and Reconstructive Surgery, Johns Hopkins School of Medicine, Baltimore, MD

## DESCRIPTION

A 72-year-old man developed tense blisters affecting 60% to 70% of his body surface area, including the torso (both lower abdomen and back) and extremities. Biopsy showed subepidermal bullous dermatosis with neutrophils and eosinophils, consistent with idiopathic immunoglobulin A (IgA) bullous dermatosis.

## QUESTIONS

1. **What causes linear IgA bullous dermatosis?**
2. **How is this condition diagnosed?**
3. **What treatments are available for this condition?**
4. **What is the prognosis?**

## DISCUSSION

Linear IgA bullous dermatosis (LABD) is a mucocutaneous autoimmune disease that may be drug-induced or idiopathic. It is characterized by linear deposition of IgA and disruption of the dermoepidermal junction, resulting in blisters with a tense clinical appearance.^1^ Mucosal surfaces with stratified squamous epithelium may also be affected. The condition has a bimodal age predilection and occurs in children between 6 months and 10 years of age, rarely persisting after puberty. Adults tend to be affected after the age of 60 years.^1^^,^^2^ When LABD occurs in adults, it may be idiopathic; however, an inciting drug may be identified. The implicated drug classes are diverse and include antibiotics, antihypertensives, and nonsteroidal anti-inflammatory agents. Vancomycin is the most commonly implicated drug.^3^ In addition, associations with lymphoproliferative disorders,^4^^,^^5^ infections,^6^^,^^7^ ulcerative colitis,^8^ and systemic lupus erythematosis^9^ have been reported.

**Figure 1:**
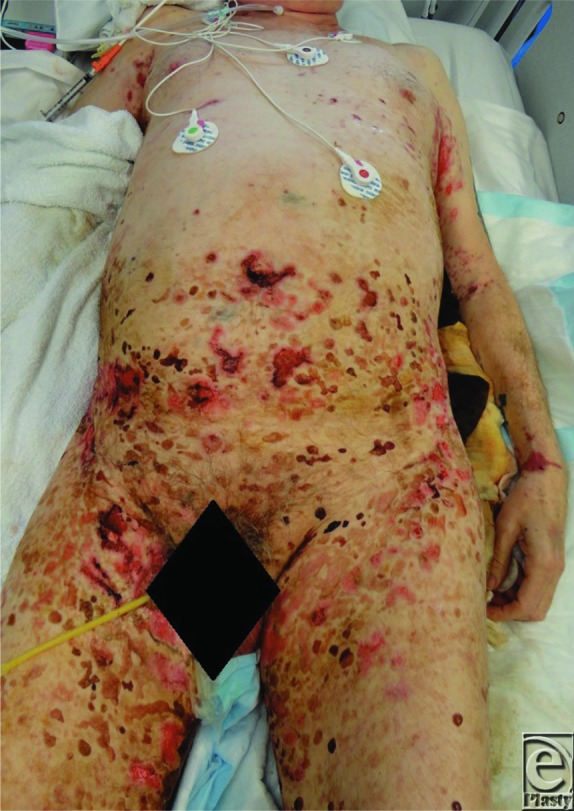
Clinical photograph of linear IgA bullous dermatosis showing tense bullae and erosions involving the lower abdomen, inner thighs, and extremities.

**Figure 2:**
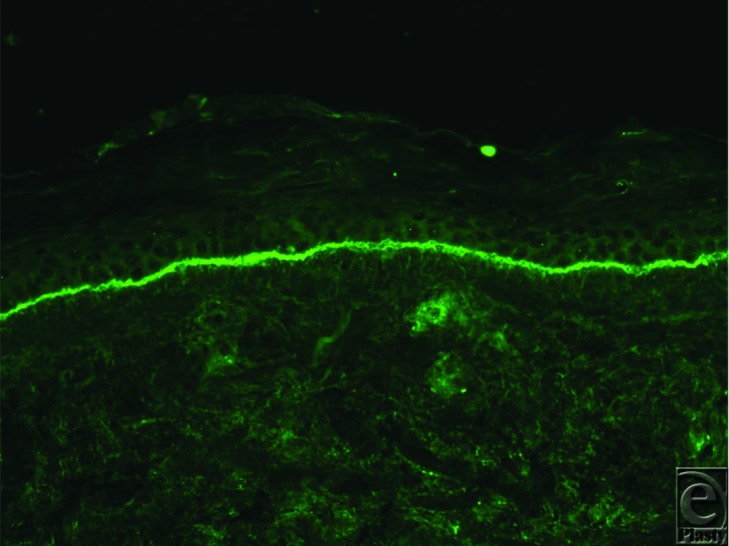
Direct immunofluorescence of linear IgA bullous dermatosis (courtesy of Grant Anhalt, MD).

Diagnosis of LABD can be aided by clinical, histopathological, and immunological data. The presence of bullae suggests bullous dermatoses. The lesions appear as clear or hemorrhagic vesicles or bullae with an erythematous or urticarial base. These are generally tense, vary in size, and form annular or circular plaques.^10^ In children, lesions are often localized to the lower abdomen, perineal area, and inner thighs (Fig 1).^2^ The face, hands, and feet are rarely involved. In adults, LABD mainly affects the extensor surfaces, trunk, buttocks, and face.^1^ Mucous membranes may be involved; the oral cavity and eyes are the most commonly affected.^11^ A skin biopsy of a lesion may show a subepithelial bulla with a predominance of neutrophils in the upper epidermis that forms papillary microabscesses. Occasionally, mononuclear cells and eosinophils may be seen.^10^ In addition, direct immunofluorescence may demonstrate the presence of IgA deposits along the basement membrane zone in a linear pattern (Fig 2). In rare circumstances, linear IgM, IgG, and C3 deposits may also be seen.^10^

The treatment of LABD varies with the degree of involvement and identification of inciting factors. When an offending drug agent is identified, withdrawal of that agent alone may result in gradual resolution of skin findings within several weeks.^12^ Therapy is largely based on case reports and case series. Dapsone, a leprostatic agent, is considered first line in the treatment of LABD. Dapsone (50-150 mg/d in adults; of note, occasionally, higher doses may be needed) may be rapidly effective in patients with LABD, and lesions may begin to resolve within 72 hours of treatment initiation.^13^ Patients who are glucose-6-phosphate dehydrogenase deficient should avoid dapsone because of the risks carried in developing severe hemolytic anemia. Complete blood cell count with differential and liver function tests should be obtained before therapy as well as during treatment. Other treatment options are less substantiated and include sulfonamides,^10^ prednisone,^11^ colchicine,^14^ tetracyclines,^15^ and nicotinamide.^16^ Systemic therapy is required until patients show clinical remission with gradual tapering toward treatment cessation.

Drug-induced LABD often resolves following cessation of the offending agent. Idiopathic LABD, however, may persist for a decade or longer with episodes of relapse and remission.^17^ Although there is no increase in mortality rate, patients with mucosal involvement may experience significant morbidity related to corneal scarring^18^ and pharyngeal and esophageal stricture formation.^19^
